# Discontinuation and Reinitiation of Dual-Labeled GLP-1 Receptor Agonists Among US Adults With Overweight or Obesity

**DOI:** 10.1001/jamanetworkopen.2024.57349

**Published:** 2025-01-31

**Authors:** Patricia J. Rodriguez, Vincent Zhang, Samuel Gratzl, Duy Do, Brianna Goodwin Cartwright, Charlotte Baker, Ty J. Gluckman, Nicholas Stucky, Ezekiel J. Emanuel

**Affiliations:** 1 Truveta Inc, Bellevue, Washington; 2 Healthcare Transformation Institute, Department of Medical Ethics and Health Policy, Perelman School of Medicine at the University of Pennsylvania, Philadelphia; 3 Center for Cardiovascular Analytics, Research and Data Science (CARDS), Providence Heart Institute, Providence Health System, Portland, Oregon

## Abstract

**Importance:**

Adherence to glucagon-like peptide-1 receptor agonists (GLP-1 RAs) is important for their effectiveness. Discontinuation and reinitiation patterns are not well understood.

**Objective:**

To describe rates of and factors associated with discontinuation and subsequent reinitiation of GLP-1 RAs among adults with overweight or obesity.

**Design, Setting, and Participants:**

In this retrospective cohort study, 125 474 adults with overweight or obesity newly initiated treatment with a dual-labeled GLP-1 RA (liraglutide, semaglutide, or tirzepatide) between January 1, 2018, and December 31, 2023, with a baseline body mass index of 27 or more, an available weight measurement within 60 days before initiation, and regular care in the year before initiation were identified using electronic health record data from a collective of US health care systems. Patients were followed up for up to 2 years to assess discontinuation and for 2 additional years to assess reinitiation.

**Exposure:**

Patients were stratified by presence of type 2 diabetes at baseline.

**Main Outcomes and Measures:**

Proportions of patients discontinuing and reinitiating GLP-1 RA were estimated from Kaplan-Meier models. Associations of sociodemographic characteristics, health factors, weight changes, and gastrointestinal adverse events with discontinuation and reinitiation outcomes were modeled using time-varying Cox proportional hazards regression models. All analyses were conducted separately for patients with and patients without type 2 diabetes.

**Results:**

In this cohort study of 125 474 adults (mean [SD] age, 54.4 [13.1] years; 82 063 women [65.4%]), 76 524 (61.0%) had type 2 diabetes. One-year discontinuation was significantly higher for patients without type 2 diabetes (64.8% [95% CI, 64.4%-65.2%]) compared with those with type 2 diabetes (46.5% [95% CI, 46.2%-46.9%]). Higher weight loss (1% reduction in weight from baseline was associated with a 3.1% [95% CI, 2.9%-3.2%] lower hazard of discontinuation for patients with type 2 diabetes and a 3.3% [95% CI, 3.2%-3.5%] lower hazard of discontinuation for patients without type 2 diabetes) and higher income (type 2 diabetes only; >$80 000: hazard ratio [HR], 0.72 [95% CI, 0.69-0.76]) were significantly associated with lower rates of discontinuation, while moderate or severe incident gastrointestinal adverse events were associated with a higher hazard of discontinuation (with type 2 diabetes: HR, 1.38 [95% CI, 1.31-1.45]; without type 2 diabetes: HR, 1.19 [95% CI, 1.12-1.27]). Of 41 792 patients who discontinued and had a discontinuation weight measurement available, 1-year reinitiation was lower for those without type 2 diabetes (36.3% [95% CI, 35.6%-37.0%]) compared with those with type 2 diabetes (47.3% [95% CI, 46.6%-48.0%]). Weight regain of 1% from discontinuation was significantly associated with increased hazards of reinitiation of 2.3% (95% CI, 1.9%-2.8%) for patients with type 2 diabetes and 2.8% (95% CI, 2.4%-3.2%) for patients without type 2 diabetes.

**Conclusions and Relevance:**

In this cohort study, most patients with overweight or obesity discontinued GLP-1 RA therapy within 1 year, but those without type 2 diabetes had higher discontinuation rates and lower reinitiation rates. Inequities in access and adherence to effective treatments have the potential to exacerbate disparities in obesity.

## Key Points

**Question:**

What are the rates of and factors associated with discontinuation and subsequent reinitiation of glucagon-like peptide-1 receptor agonists (GLP-1 RAs) among adults with overweight or obesity?

**Findings:**

In this cohort study of 125 474 patients initiating GLP-1 RAs, 46.5% of patients with and 64.8% without type 2 diabetes discontinued within 1 year; 47.3% of patients with and 36.3% without type 2 diabetes subsequently reinitiated a GLP-1 RA within 1 year. Weight loss, income, and adverse events were significantly associated with discontinuation, while weight regain was significantly associated with reinitiation.

**Meaning:**

This study suggests that although most patients discontinue GLP-1 RAs, discontinuation rates are significantly higher and reinitiation rates are significantly lower for patients without type 2 diabetes.

## Teaser

This cohort study describe rates of and factors associated with discontinuation and subsequent reinitiation of glucagon-like peptide-1 receptor agonists among more than 125 000 adults with overweight or obesity.

## Introduction

More than 73% of US adults have obesity or overweight,^1^ incurring approximately $173 billion in associated medical costs annually.^2^ Although glucagon-like peptide-1 receptor agonists (GLP-1 RAs) substantially lower weight,^3,4,5,6,7^ hemoglobin A_1c_ levels, and cardiovascular risk,^8^ trials suggest they must be continued for sustained effects.^9,10^

Ongoing use of GLP-1 RAs may be limited by issues related to tolerability, efficacy, access, and cost.^11^ More than 70% of patients in clinical trials of GLP-1 RAs reported adverse events, most often gastrointestinal (GI), that were mild.^3,4,5,6,7^ Although most patients taking a GLP-1 RA achieve clinically meaningful weight loss, heterogeneity in response exists.^3,4,5,6,7,12^ In addition, most patients’ weight loss plateaus.^5,6,13^ Accordingly, a lack of desired weight loss could lead to discontinuation of GLP-1 RAs for some. Because obesity is more prevalent among individuals with low income, access and affordability to GLP-1 RAs may represent a major barrier to initiation and continued use.^14,15^ In addition, limited payer coverage of antiobesity medications and off-label use of antidiabetes medications, including GLP-1 RAs, still represents a major challenge for many patients.^16,17,18^ Finally, shortages of GLP-1 RAs may limit continued use.^19^

High and widely varying rates of GLP-1 RA discontinuation at 1 year have been reported, ranging from 37% to 81%.^20,21,22,23,24^ Multiple factors have been associated with discontinuation, including cost, insurance type, comorbidities, and absence of type 2 diabetes.^22,23^ The magnitude of weight loss (and its association with GLP-1 RA discontinuation) has largely been omitted from previous analyses. In addition, patterns of reinitiation after discontinuation have been poorly characterized. Because the same factors associated with discontinuation may also be associated with reinitiation, further insights are needed.

This study describes the rates of and factors associated with discontinuation of GLP-1 RAs and dual gastric inhibitory polypeptide receptor–GLP-1 RAs labeled for both type 2 diabetes and obesity (liraglutide, semaglutide, and tirzepatide) in adults with or without type 2 diabetes. The study further describes the rates of and factors associated with reinitiation of these medications. The role of time-varying weight change was included in both analyses.

## Methods

### Study Design

New users of liraglutide, injectable semaglutide, or tirzepatide (henceforth referred to as *GLP-1 RAs*) with overweight or obesity (regardless of type 2 diabetes) between January 1, 2018, and December 31, 2023, were included. New users were defined as those never previously dispensed any GLP-1 RA, based on all available history. Patients 18 years of age or older with a baseline body mass index (BMI; calculated as weight in kilograms divided by height in meters squared) of 27 or higher, with baseline weight measurement available, regular interactions with the health care system in the previous year, and a prescription for a GLP-1 RA in the preceding 2 months were included. Patients were followed up for up to 2 years to assess discontinuation. Patients who discontinued their GLP-1 RA and had a weight measurement available within 60 days before discontinuation were followed up for up to 2 additional years to assess reinitiation of any GLP-1 RA. This study used only deidentified patient records and therefore did not require institutional review board approval or patient consent in accordance with the Common Rule. Deidentification is attested to through expert determination in accordance with the Health Insurance Portability and Accountability Act Privacy Rule. This retrospective observational cohort study followed the Strengthening the Reporting of Observational Studies in Epidemiology (STROBE) reporting guideline.^25^

### Data Source

This study used a subset of Truveta Data. Truveta provides access to continuously updated and linked electronic health record (EHR) data from a collective of 30 US health care systems. This includes data on demographic characteristics, encounters, diagnoses, vital signs (eg, weight, BMI, and blood pressure), medication requests (prescriptions), and laboratory and diagnostic tests and results (eg, hemoglobin A_1c_ tests and values). Concepts from unstructured clinical notes, including reasons for medication discontinuation, are extracted using natural language processing. Updated EHR data are provided daily to Truveta by constituent health care systems. In addition to EHR data for care delivered within Truveta constituent health care systems, data on medication dispensing and social determinants of health were made available through linked third-party data. Medication dispensing (via eprescribing) includes fills for prescriptions written both within and outside Truveta constituent health care systems, resulting in greater availability of patients’ medication history. Medication dispensing histories are updated at the time of the encounter and include fill dates, National Drug Codes or RxNorm codes, quantity dispensed, and number of days of medication supplied. Social determinants of health data include individual-level factors, including income and education. Income data represent estimated individual income, sourced by LexisNexis Risk solutions from a variety of public and proprietary data sources, including property data, tax-assessment records, registrations, bankruptcies, and education data.^26^ Truveta presents income values as a range to obscure the identity of the individual and protect the privacy of the dataset.

Data are normalized into a common data model through syntactic and semantic normalization. Truveta data are then deidentified; once deidentified, data are available for analysis in R, version 4.4.1 (R Project for Statistical Computing), or Python, version 3.10.13 (Python Software Foundation), using Truveta Studio. Data for this study were accessed on November 5, 2024.

### Study Population

We identified adults with overweight or obesity first dispensed a GLP-1 RA between January 1, 2018, and December 31, 2023. We required a BMI of 27 or higher and a weight measured in the 60 days preceding the first dispensing of a GLP-1 RA. To improve outcome observability, we restricted the analysis to patients receiving usual care within a Truveta health care system in the prior year, defined as an interaction in both consecutive 6-month periods in the year before GLP-1 RA initiation. Patients with a history of type 1 diabetes, gestational diabetes, or diabetic retinopathy, as well as those with missing data on sex or with no follow-up encounters after GLP-1 RA initiation were excluded. Reinitiation analyses were further restricted to patients who discontinued a GLP-1 RA within 2 years, for whom there was an encounter after GLP-1 RA discontinuation and an available weight measurement taken up to 60 days before to 1 day after discontinuation of the GLP-1 RA.

The first GLP-1 RA dispensing was considered the initiation date, and the first GLP-1 RA dispensed was considered the index medication. Brand names were used to classify index medications as antiobesity medications or antidiabetes medications. Patients were classified as having type 2 diabetes if they had a diagnosis code for type 2 diabetes, used insulin or a dipeptidyl peptidase 4 (DPP-4) inhibitor, or had a hemoglobin A_1c_ level higher than 7.5% in the 2 years before GLP-1 RA initiation.

### Outcomes

Outcomes of primary discontinuation and subsequent reinitiation were considered separately. Primary discontinuation was defined as the first date a patient was 60 days or more without any GLP-1 RA on hand, using fill dates and number of days of medication supplied to account for excess (carryover) medication from previous fills. Fills of all GLP-1 RAs were considered; GLP-1 RAs dispensed on subsequent fills were not required to match the index GLP-1 RA brand. Reinitiation was defined as the first fill of any GLP-1 RA after primary discontinuation. Sensitivity analyses were conducted using an alternative window of 90 days or more for discontinuation (eResults in Supplement 1).

Both discontinuation and reinitiation were treated as time-to-event outcomes to account for censoring. Patients were followed up until the first outcome occurrence, the last encounter before study end (November 5, 2024), or 2 years from the index event. In discontinuation analyses, the first GLP-1 RA dispensing served as the index event. In reinitiation analyses, the first discontinuation date served as the index event.

Associations of discontinuation with safety, tolerability, effectiveness, health factors, sociodemographic characteristics, and access were considered. The variables included were selected a priori based on existing evidence and hypothesized associations. Safety and tolerability were modeled as the on-treatment time-varying presence of a moderate or severe GI adverse event (bowel obstruction, cholecystitis, cholelithiasis, gastroenteritis, gastroparesis, or pancreatitis). Mild adverse events, such as nausea and vomiting, were not included given the expectation of inconsistent EHR capture. Effectiveness was modeled as the on-treatment time-varying change in weight relative to baseline, where baseline was the most recent weight in the 60 days before initiation. For interpretability, this was modeled per 1% weight loss. Weight change values were updated each time a new weight value was available. Weight cleaning approaches have been described elsewhere.^12^ Health factors included baseline BMI, along with presence of chronic kidney disease and/or heart failure at baseline. Separate models were used for patients with or without type 2 diabetes, given the expectation of different associations between covariates and discontinuation outcomes. Sociodemographic and access factors included sex, age, race and ethnicity reported in the health system, age 65 years or older (a proxy for Medicare age eligibility), and individual income. Race and ethnicity documented in the EHR may have been determined using patient-reported information or clinician ascertainment. Race and ethnicity were included in the study given the expectation that, as correlates of social determinants of health, discontinuation and reinitiation may differ between racial and ethnic groups. Insurance status was not available. Baseline hazards were stratified by year of initiation to account for differences in marginal discontinuation and proportionality over time. For the subset of patients with clinical notes data available, we describe the reasons for GLP-1 RA discontinuation from extracted clinical notes (eMethods and eFigure 5 in Supplement 1).

For those who discontinued their GLP-1 RA, reinitiation analyses considered the same factors as for discontinuation, but used time-invariant variables for the on-treatment presence of any GI adverse event and the total on-treatment weight change (change from baseline to discontinuation). Duration of initial treatment (in months) was included as a time-invariant variable. Time-varying weight change since discontinuation was included to consider the potential association with outcomes of weight regain since the GLP-1 RA was stopped. For interpretability, this was modeled per 1% weight gain since discontinuation. Weight change values were updated each time a new weight value was available, with changes reported relative to the discontinuation weight.

### Statistical Analysis

The proportion of at-risk patients who discontinued and subsequently reinitiated treatment with a GLP-1 RA within 2 years were extracted from separate Kaplan-Meier models stratified by the presence of type 2 diabetes at initiation. A sensitivity analysis was performed to evaluate patients for whom 2 years of follow-up were fully available. Potential factors associated with outcomes of discontinuation and reinitiation for those with or without type 2 diabetes were estimated using separate time-varying Cox proportional hazards regression models inclusive of the variables described. Additional information on statistical methods is provided in the eMethods and eFigures 1 and 2 in Supplement 1. All *P* values were from 2-sided tests, and results were deemed statistically significant at *P* < .05.

## Results

Overall, 125 474 adults met inclusion criteria, including 76 524 patients (61.0%) with type 2 diabetes (75 434 [98.6%] using antidiabetes medications and 1090 [1.4%] using antiobesity medications) and 48 950 patients (39.0%) without type 2 diabetes (30 236 [61.8%] using antidiabetes medications off-label and 18 714 [38.2%] using antiobesity medications) (Table 1). The mean (SD) age overall was 54.4 (13.1) years; 82 063 patients (65.4%) were female, and 43 411 patients (34.6%) were male; and 2647 patients (2.1%) were Asian, 18 238 (14.5%) were Black or African American, 16 095 (12.8%) were Hispanic, 92 263 (73.5%) were White, and 12 326 (9.8%) were other or unknown race or ethnicity (Alaska Native or American Indian, Hative Hawaiian or Pacific Islander, multiple races, other single races, and unknown or missing race). Among patients with income information (105 841 [84.4%]), individual income exceeded $50 000 for 33 742 of 64 650 (52.2%) with type 2 diabetes and 24 291 of 41 191 (59.0%) without type 2 diabetes. The median BMI at baseline was 37.3 (IQR, 33.1-42.7), with 45 751 patients (36.5%) overall classified as having class 3 obesity (BMI ≥40).

**Table 1.** Characteristics of Patients Initiating GLP-1 RAs Meeting Study Criteria

Characteristic	Patients, No. (%)		
	Type 2 diabetes (n = 76 524)	No type 2 diabetes (n = 48 950)	Overall (N = 125 474)
Age group, y			
18-44	12 249 (16.0)	18 258 (37.3)	30 507 (24.3)
45-64	42 191 (55.1)	24 074 (49.2)	66 265 (52.8)
≥65	22 084 (28.9)	6618 (13.5)	28 702 (22.9)
Sex			
Female	43 380 (56.7)	38 683 (79.0)	82 063 (65.4)
Male	33 144 (43.3)	10 267 (21.0)	43 411 (34.6)
Race			
Asian	1813 (2.4)	834 (1.7)	2647 (2.1)
Black or African American	11 301 (14.8)	6937 (14.2)	18 238 (14.5)
White	55 357 (72.3)	36 906 (75.4)	92 263 (73.5)
Other or unknown^a^	8053 (10.5)	4273 (8.7)	12 326 (9.8)
Ethnicity			
Hispanic or Latino	10 477 (13.7)	5618 (11.5)	16 095 (12.8)
Not Hispanic or Latino	62 506 (81.7)	41 244 (84.3)	103 750 (82.7)
Other or unknown^b^	3541 (4.6)	2088 (4.3)	5629 (4.5)
Education: some college on record	29 637 (38.7)	29 240 (59.7)	58 877 (46.9)
Individual income, $			
≤30 000	4456 (5.8)	2823 (5.8)	7279 (5.8)
30 001-50 000	26 452 (34.6)	14 077 (28.8)	40 529 (32.3)
50 001-80 000	24 916 (32.6)	16 580 (33.9)	41 496 (33.1)
>80 000	8826 (11.5)	7711 (15.8)	16 537 (13.2)
Unknown	11 874 (15.5)	7759 (15.9)	19 633 (15.6)
Index medication			
Liraglutide	9918 (13.0)	8053 (16.5)	17 971 (14.3)
Semaglutide	56 680 (74.1)	33 788 (69.0)	90 468 (72.1)
Tirzepatide	9926 (13.0)	7109 (14.5)	17 035 (13.6)
Index medication label			
Antidiabetic medication	75 434 (98.6)	30 236 (61.8)	105 670 (84.2)
Antiobesity medication	1090 (1.4)	18 714 (38.2)	19 804 (15.8)
Index year			
2018	2916 (3.8)	611 (1.2)	3527 (2.8)
2019	5365 (7.0)	1101 (2.2)	6466 (5.2)
2020	4246 (5.5)	1185 (2.4)	5431 (4.3)
2021	8199 (10.7)	3937 (8.0)	12 136 (9.7)
2022	17 362 (22.7)	15 379 (31.4)	32 741 (26.1)
2023	38 436 (50.2)	26 737 (54.6)	65 173 (51.9)
Baseline BMI, median (IQR)	37.1 (32.9-42.6)	37.6 (33.4-43.0)	37.3 (33.1-42.7)
Baseline BMI class			
Overweight: 27-30	7743 (10.1)	3530 (7.2)	11 273 (9.0)
Obesity class 1 (30 to <35)	21 109 (27.6)	13 268 (27.1)	34 377 (27.4)
Obesity class 2 (35 to <40)	20 430 (26.7)	13 643 (27.9)	34 073 (27.2)
Obesity class 3 (≥40)	27 242 (35.6)	18 509 (37.8)	45 751 (36.5)
Baseline weight, median (IQR), kg	107.0 (92.5-124.7)	104.8 (90.7-122.2)	106.1 (91.7-123.8)
Comorbidities			
Atrial fibrillation	5707 (7.5)	1874 (3.8)	7581 (6.0)
Asthma	14 746 (19.3)	9707 (19.8)	24 453 (19.5)
COPD	6580 (8.6)	1824 (3.7)	8404 (6.7)
Chronic kidney disease	17 015 (22.2)	3872 (7.9)	20 887 (16.6)
Glaucoma	2086 (2.7)	596 (1.2)	2682 (2.1)
Heart failure	7112 (9.3)	1627 (3.3)	8739 (7.0)
Hypertension	61 722 (80.7)	24 637 (50.3)	86 359 (68.8)
Hyperlipidemia	62 659 (81.9)	24 929 (50.9)	87 588 (69.8)
Ischemic heart disease	7606 (9.9)	1718 (3.5)	9324 (7.4)
Major depressive disorder	13 783 (18.0)	9574 (19.6)	23 357 (18.6)
Medications			
Metformin	60 648 (79.3)	10 676 (21.8)	71 324 (56.8)
Insulin	7109 (9.3)	0 (0.0)	7109 (5.7)
DPP-4 inhibitor	13 564 (17.7)	0 (0.0)	13 564 (10.8)
SGLT2 inhibitor	21 883 (28.6)	1434 (2.9)	23 317 (18.6)
Sulfonylurea	23 259 (30.4)	662 (1.4)	23 921 (19.1)
Orlistat	108 (0.1)	218 (0.4)	326 (0.3)
Phentermine-topiramate	209 (0.3)	806 (1.6)	1015 (0.8)

Abbreviations: BMI, body mass index (calculated as weight in kilograms divided by height in meters squared); COPD, chronic obstructive pulmonary disease; DPP-4, dipeptidyl peptidase-4; GLP-1 RA, glucagon-like peptide-1 receptor agonist; SGLT2, sodium-glucose cotransporter 2.

^a^ Includes Alaska Native or American Indian, Native Hawaiian or Pacific Islander, multiple races, other single races, and unknown or missing race.

^b^ Includes values other than Hispanic or Latino, not Hispanic or Latino, and unknown or missing ethnicity.

The median time between GLP-1 RA initiation and censoring (last encounter before November 5, 2024, or 2 years, whichever occurred first) was 618 days (IQR, 470-730 days). Overall, 112 634 patients (89.8%) were followed up for at least 1 year, and 48 099 (38.3%) for at least 2 years after GLP-1 RA initiation.

### Discontinuation

**Figure 1.**
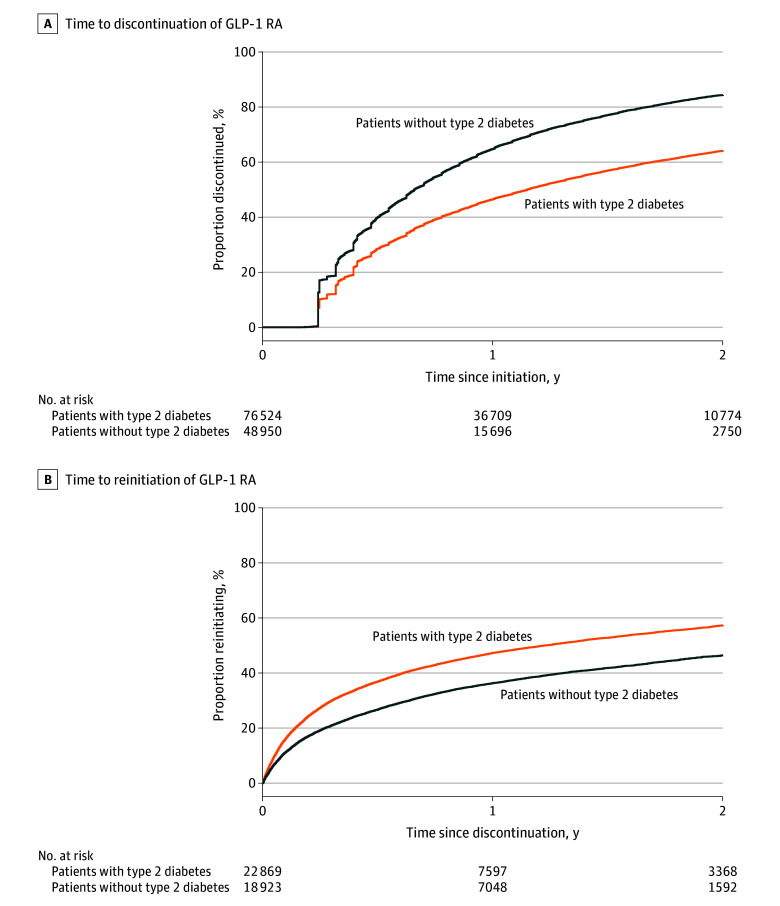
Proportions Discontinuing and Reinitiating Glucagon-Like Peptide-1 Receptor Agonist (GLP-1 RA) Within 2 Years Discontinuation is the first date a patient is 60 days or more without any GLP-1 RA medication on hand. Reinitiation is the first fill of any GLP-1 RA after discontinuation. Patients were censored at their last encounter before November 5, 2024, or administrative censoring (2 years), whichever occurred first. The y-axis represents the event probability (1-survival probability).

In total, 81 919 patients discontinued their GLP-1 RA within 2 years. Accounting for censoring, 53.6% (95% CI, 53.4%-53.9%) discontinued by 1 year, and 72.2% (95% CI, 71.9%-72.4%) discontinued by 2 years. Discontinuation rates were significantly lower for patients with type 2 diabetes (46.5% [95% CI, 46.2%-46.9%] by 1 year and 64.1% [95% CI, 63.7%-64.5%] by 2 years) compared with patients without type 2 diabetes (64.8% [95% CI, 64.4%-65.2%] by 1 year and 84.4% [95% CI, 84.0%-84.8%] by 2 years) (Figure 1A). In a sensitivity analysis limited to those followed up for 2 full years, similar discontinuation rates were observed in a sensitivity analysis limited to those followed up for 2 full years (53.6% [95% CI, 53.2%-54.0%] discontinued by 1 year and 71.9% [95% CI, 71.5%-72.3%] by 2 years).

**Figure 2.**
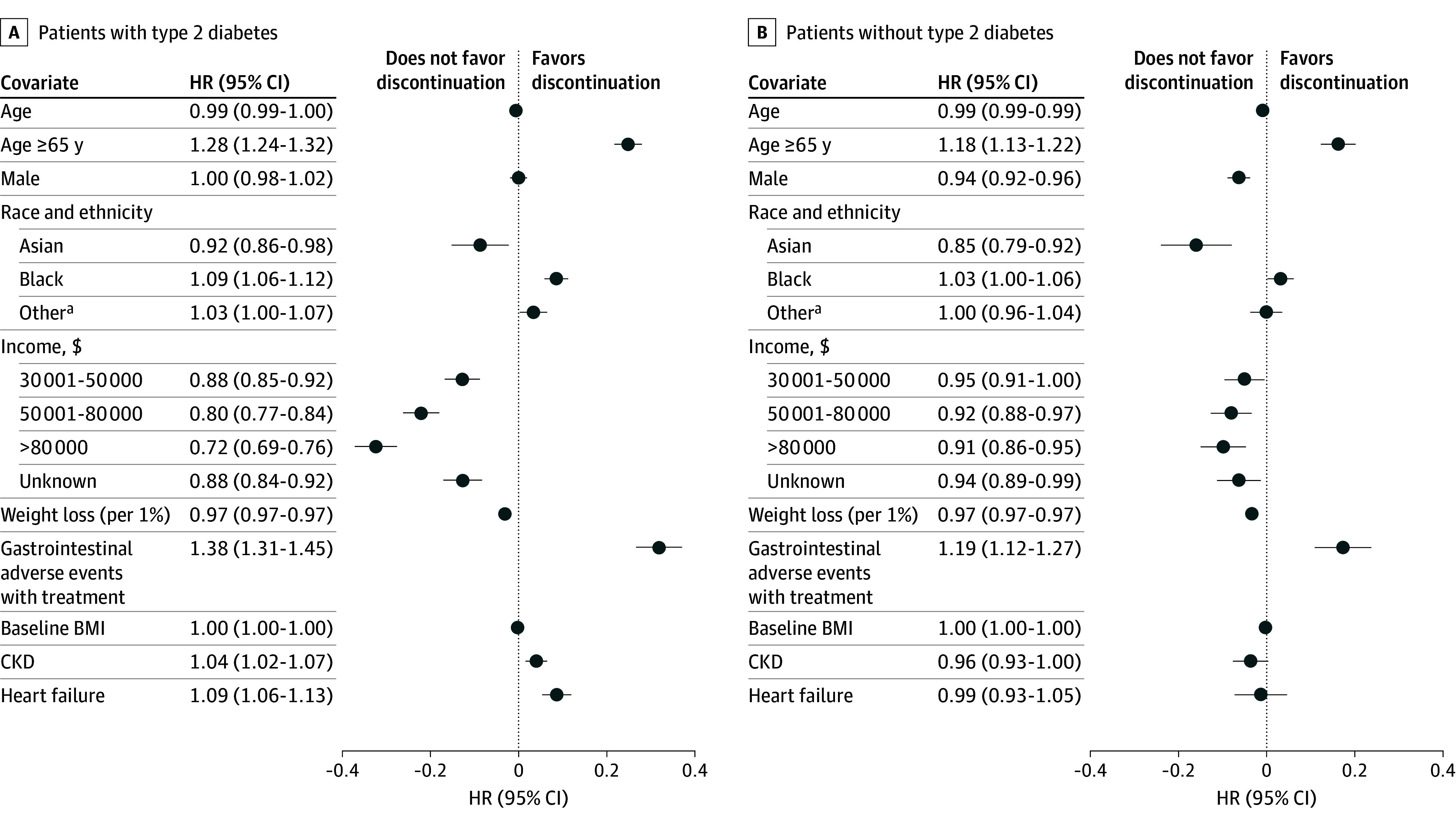
Associations Between Covariates and Discontinuation Outcomes for Patients With or Without Type 2 Diabetes Points represent hazard ratios (HRs) from separate Cox proportional hazards regression models for patients with or without type 2 diabetes, plotted on a log scale. Lines represent 95% CIs, plotted on a log scale. Tabular HRs (95% CIs) are provided on an exponentiated (HR) scale. The comparator reference level for sex was female, for race and ethnicity was White, and for income was $30 000 or less. Baseline hazards were stratified by initiation year. BMI indicates body mass index; CKD, chronic kidney disease. ^a^Includes Alaska Native or American Indian, Hawaiian or Pacific Islander, multiple races, other single races, and unknown or missing race.

Several factors were significantly associated with discontinuation (Figure 2). Patients 65 years of age or older were more likely to discontinue their GLP-1 RA (with type 2 diabetes: hazard ratio [HR], 1.28 [95% CI, 1.24-1.32]; without type 2 diabetes: HR, 1.18 [95% CI, 1.13-1.22]). Higher income was progressively associated with a lower rate of discontinuation among those with type 2 diabetes. Compared with those with incomes under $30 000, HRs for discontinuation were 0.88 (95% CI, 0.85-0.92) for those with incomes of $30 001 to $50 000, 0.80 (95% CI, 0.77-0.84) for those with incomes of $50 001 to $80 000, and 0.72 (95% CI, 0.69-0.76) for those with incomes of more than $80 000. Higher on-treatment weight loss was also significantly associated with lower discontinuation. A 1% reduction in weight from baseline was associated with a 3.1% (95% CI, 2.9%-3.2%) lower hazard of discontinuation for patients with type 2 diabetes and a 3.3% (95% CI, 3.2%-3.5%) lower hazard of discontinuation for patients without type 2 diabetes. Finally, on-treatment moderate or severe incident GI adverse events were associated with a higher hazard of discontinuation for patients with type 2 diabetes (HR, 1.38 [95% CI, 1.31-1.45]) and for patients without type 2 diabetes (HR, 1.19 [95% CI, 1.12-1.27]). Results were similar using a 90-day discontinuation window (eFigures 3 and 4 in Supplement 1). Adverse effects and cost were the most frequent specific reasons for discontinuation documented in clinical notes (eFigure 5 in Supplement 1).

### Reinitiation

Of 81 919 patients who discontinued their GLP-1 RA, 41 792 (51.0%) were included in reinitiation analyses, based on having a weight recorded at discontinuation and at 1 or more encounters after discontinuation. The median duration of primary treatment with a GLP-1 RA was 176 days (IQR, 116-306 days), with a median on-treatment weight change of −2.9% (IQR, −6.9% to 0.0%). The population evaluated for reinitiation was mostly female, White, and not Hispanic or Latino (Table 2). After primary discontinuation, the median follow-up time was 435 days (IQR, 271-730 days), with 25 483 of 41 792 patients (61.0%) followed up for at least 1 year and 10 483 of 41 792 patients (25.1%) followed up for at least 2 years.

**Table 2.** Characteristics of Discontinued Patients Who Met Additional Study Criteria and Who Were Included in Analyses of Reinitiation

Characteristic	Patients, No. (%)		
	Type 2 diabetes (n = 22 869)	No type 2 diabetes (n = 18 923)	Overall (N = 41 792)
Age group, y			
18-44	3475 (15.2)	7065 (37.3)	10 540 (25.2)
45-64	11 367 (49.7)	8923 (47.2)	20 290 (48.5)
≥65	8027 (35.1)	2935 (15.5)	10 962 (26.2)
Sex			
Female	13 478 (58.9)	15 319 (81.0)	28 797 (68.9)
Male	9391 (41.1)	3604 (19.0)	12 995 (32.1)
Race			
Asian	409 (1.8)	270 (1.4)	679 (1.6)
Black or African American	3523 (15.4)	2836 (15.0)	6359 (15.2)
White	16 618 (72.7)	14 175 (74.9)	30 793 (73.7)
Other or unknown^a^	2319 (10.1)	1642 (8.7)	3961 (9.5)
Ethnicity			
Hispanic or Latino	3079 (13.5)	2256 (11.9)	5335 (12.8)
Not Hispanic or Latino	18 784 (82.1)	15 951 (84.3)	34 735 (83.1)
Other or unknown^b^	1006 (4.4)	716 (3.8)	1722 (4.1)
Education: some college on record	7961 (34.8)	11 097 (58.6)	19 058 (45.6)
Individual income, $			
≤30 000	1597 (7.0)	1242 (6.6)	2839 (6.8)
30 001-50 000	8379 (36.6)	5825 (30.8)	14 204 (34.0)
50 001-80 000	6953 (30.4)	6143 (32.5)	13 096 (31.3)
>80 000	2222 (9.7)	2708 (14.3)	4930 (11.8)
Unknown	3718 (16.3)	3005 (15.9)	6723 (16.1)
Index medication			
Liraglutide	3868 (16.9)	3529 (18.6)	7397 (17.7)
Semaglutide	16 742 (73.2)	12 682 (67.0)	29 424 (70.4)
Tirzepatide	2259 (9.9)	2712 (14.3)	4971 (11.9)
Index medication label			
Antidiabetic medication	22 382 (97.9)	11 706 (61.9)	34 088 (81.6)
Antiobesity medication	487 (2.1)	7217 (38.1)	7704 (18.4)
Baseline BMI, median (IQR)	37.1 (32.8 to 42.7)	37.9 (33.6 to 43.4)	37.5 (33.1 to 43.0)
Baseline BMI class			
Overweight: 27-30	2458 (10.7)	1293 (6.8)	3751 (9.0)
Obesity Class 1 (30 to <35)	6225 (27.2)	4963 (26.2)	11 188 (26.8)
Obesity Class 2 (35 to <40)	6011 (26.3)	5221 (27.6)	11 232 (26.9)
Obesity Class 3 (≥40)	8175 (35.7)	7446 (39.3)	15 621 (37.4)
Duration of initial treatment, median (IQR), d	177.0 (116.0 to 310.0)	174.0 (116.0 to 302.0)	176.0 (116.0 to 306.0)
Weight at initiation, median (IQR), kg	106.2 (91.6 to 124.1)	105.1 (90.7 to 122.7)	105.7 (91.2 to 123.4)
Weight at discontinuation, median, (IQR), kg	103.0 (88.5 to 120.2)	99.8 (85.3 to 117.9)	101.6 (86.8 to 119.3)
Weight loss on treatment, median (IQR), %	2.3 (−0.1 to 5.8)	3.7 (0.4 to 8.5)	2.9 (0.0 to 6.9)
Presence of GI adverse event on treatment	1050 (4.6)	655 (3.5)	1705 (4.1)
Comorbidities			
Atrial fibrillation	2110 (9.2)	815 (4.3)	2925 (7.0)
Asthma	5005 (21.9)	4098 (21.7)	9103 (21.8)
COPD	2619 (11.5)	869 (4.6)	3488 (8.3)
Chronic kidney disease	6220 (27.2)	1693 (8.9)	7913 (18.9)
Glaucoma	748 (3.3)	241 (1.3)	989 (2.4)
Heart failure	2858 (12.5)	766 (4.0)	3624 (8.7)
Hypertension	18 955 (82.9)	9919 (52.4)	28 874 (69.1)
Hyperlipidemia	18 913 (82.7)	9841 (52.0)	28 754 (68.8)
Ischemic heart disease	2903 (12.7)	755 (4.0)	3658 (8.8)
Major depressive disorder	4819 (21.1)	4212 (22.3)	9031 (21.6)
Medications			
Metformin	17 582 (76.9)	3767 (19.9)	21 349 (51.1)
Insulin	2727 (11.9)	0 (0.0)	2727 (6.5)
DPP-4 inhibitor	4056 (17.7)	0 (0.0)	4056 (9.7)
SGLT2 inhibitor	6683 (29.2)	485 (2.6)	7168 (17.2)
Sulfonylurea	7489 (32.7)	208 (1.1)	7697 (18.4)
Orlistat	43 (0.2)	95 (0.5)	138 (0.3)
Phentermine-topiramate	64 (0.3)	296 (1.6)	360 (0.9)

Abbreviations: BMI, body mass index (calculated as weight in kilograms divided by height in meters squared); COPD, chronic obstructive pulmonary disease; DPP-4, dipeptidyl peptidase 4; GI, gastrointestinal; SGLT2, sodium-glucose cotransporter 2.

^a^ Includes Alaska Native or American Indian, Hawaiian or Pacific Islander, multiple races, other single races, and unknown or missing race.

^b^ Includes values other than Hispanic or Latino, not Hispanic or Latino, and unknown or missing ethnicity.

Accounting for censoring, 47.3% (95% CI, 46.6%-48.0%) of patients with type 2 diabetes and 36.3% (95% CI, 35.6%-37.0%) of patients without type 2 diabetes reinitiated a GLP-1 RA within 1 year, and 57.3% (95% CI, 56.5%-58.1%) with type 2 diabetes and 46.4% (95% CI, 45.4%-47.4%) without type 2 diabetes reinitiated a GLP-1 RA within 2 years of discontinuation (Figure 1B).

**Figure 3.**
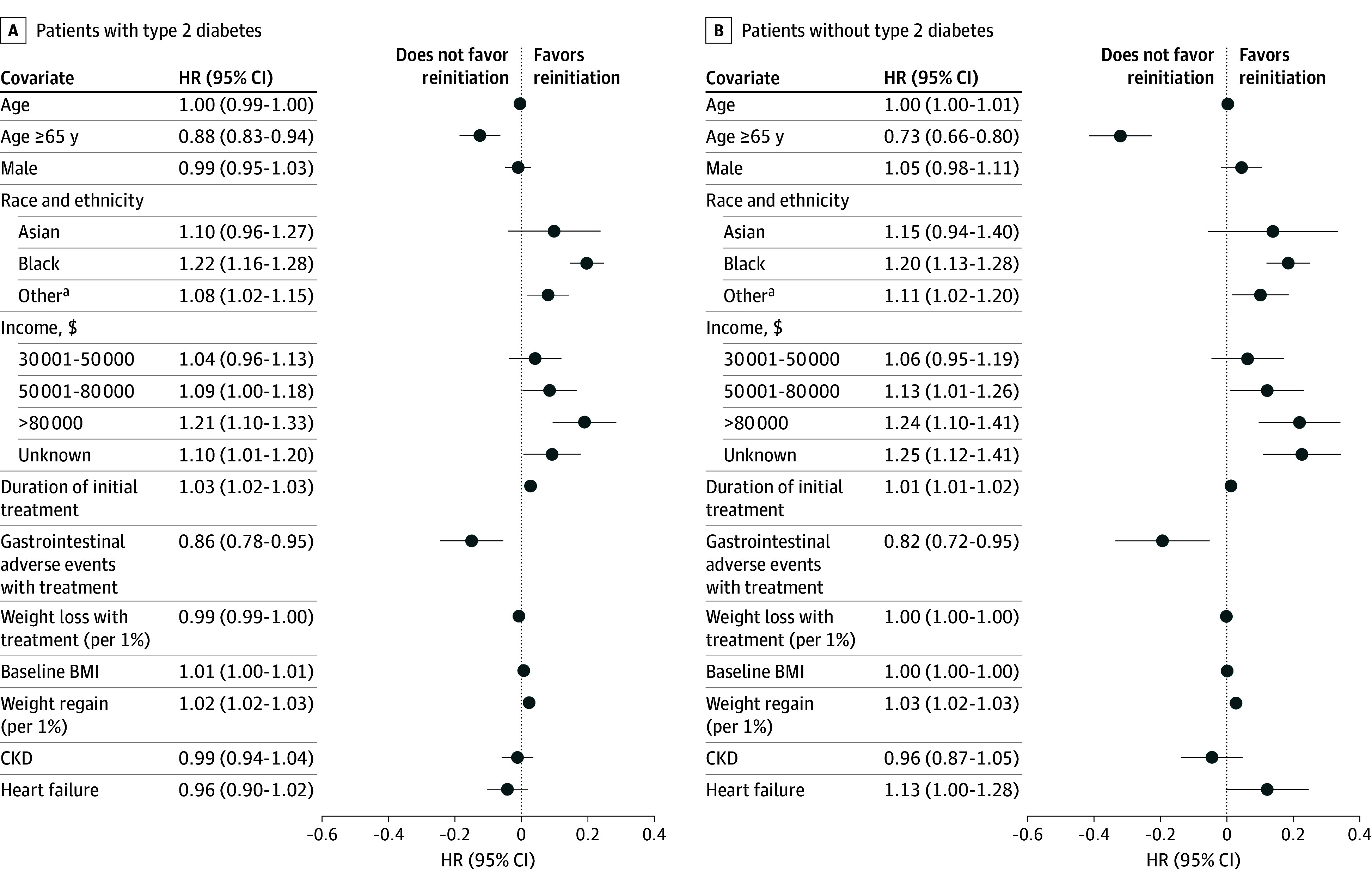
Associations Between Covariates and Reinitiation Outcomes for Patients With or Without Type 2 Diabetes Points represent hazard ratios (HRs) from separate Cox proportional hazards regression models for patients with or without type 2 diabetes, plotted on a log scale. Lines represent 95% CIs, plotted on a log scale. Tabular HRs (95% CIs) are provided on an exponentiated (HR) scale. The comparator reference level for sex was female, for race and ethnicity was White, and for income was $30 000 or less. Baseline hazards were stratified by initiation year. BMI indicates body mass index; CKD, chronic kidney disease. ^a^Includes Alaska Native or American Indian, Hawaiian or Pacific Islander, multiple races, other single races, and unknown or missing race.

Several factors were associated with reinitiation (Figure 3). Those aged 65 years or older were significantly less likely to reinitiate their GLP-1 RA (with type 2 diabetes: HR, 0.88 [95% CI, 0.83-0.94]; without type 2 diabetes: HR, 0.73 [95% CI, 0.66-0.80]). Although associations between income and reinitiation were not consistently statistically significant in either group, point estimates suggested a positive correlation between higher income and higher reinitiation. In addition, a 1% weight gain since discontinuation was significantly associated with a 2.3% (95% CI, 1.9%-2.8%) increased hazard of reinitiation for patients with type 2 diabetes and a 2.8% (95% CI, 2.4%-3.2%) increased hazard of reinitiation for patients without type 2 diabetes. Finally, moderate or severe GI adverse events during initial treatment were associated with a lower rate of reinitiation for patients with type 2 diabetes (HR, 0.86 [95% CI, 0.78-0.95]) and for patients without type 2 diabetes (HR, 0.82 [95% CI, 0.72-0.95]).

## Discussion

Most patients who newly initiated treatment with GLP-1 RA discontinued treatment within 1 year, with a significantly higher rate among patients without type 2 diabetes. These same patients (without type 2 diabetes) also had a significantly lower rate of GLP-1 RA reinitiation. Greater on-treatment weight loss was associated with a lower likelihood of GLP-1 RA discontinuation; similarly, weight gain since discontinuation was associated with an increased likelihood of GLP-1 RA reinitiation.

Three points need emphasis. First, the high rate of GLP-1 RA discontinuation observed in this study (53.6% by 1 year) is consistent with other analyses. Recent studies have found discontinuation rates ranging from 37% to 81%, depending on the population, data source, and time period considered.^20,21,22,23,24,27^ The finding of an increased discontinuation rate for GLP-1 RAs among patients without type 2 diabetes and with adverse GI adverse events is also similar to that noted in previous studies.^23^

Second, the associations between weight loss and discontinuation and between weight regain and reinitiation suggest that weight management is an important factor regardless of type 2 diabetes status. Third, lower income was also associated with a higher likelihood of discontinuation among those with type 2 diabetes but not those without type 2 diabetes. Patients without type 2 diabetes had higher baseline incomes. Those highly sensitive to affordability may have declined to initiate GLP-1 RAs in the first place, given limited insurance coverage for patients without type 2 diabetes. In addition, lower-cost treatment alternatives, such as metformin or DPP-4 inhibitors, are available for patients with type 2 diabetes.^28^ In contrast, the lack of effective alternative treatments for patients without type 2 diabetes may necessitate GLP-1 RA treatment regardless of cost. The importance of cost in discontinuation was also reflected in clinical notes data. Further studies should investigate the association of insurance coverage and out-of-pocket costs with GLP-1 RA initiation and adherence.

Our findings have important policy implications associated with equitable access and outcomes for patients with or without type 2 diabetes. Lack of medication adherence may limit the long-term health benefits, such as cardiovascular risk reduction, associated with GLP-1 RA therapy. Our analysis suggests greater efforts are needed to increase access and adherence for patients without type 2 diabetes and those with lower incomes.

### Strengths and Limitations

This study has several strengths. To our knowledge, this is the first study to characterize patterns of reinitiation after GLP-1 RA discontinuation. Use of EHR data also allowed for inclusion of weight changes during treatment and after discontinuation. Given limited insurance coverage of antiobesity medications, use of linked EHR and dispensing information allowed for the inclusion of patients paying for GLP-1 RAs out of pocket. Last, use of survival models, instead of binary outcomes models, allowed for the use of very recent data that were subject to high censoring.

This study also has several limitations. First, GLP-1 RA shortages were not modeled explicitly in this analysis, given insufficient data. Although inclusion of strata for the year of initiation may account for some differences in medication availability over time, it is unlikely to fully characterize shortage-related associations. Second, discontinuation definitions in the literature vary and are likely subject to sensitivity vs specificity trade-offs. Although we defined discontinuation as 60 days without any medication on hand based on both clinical and data considerations, some patients may have been misclassified. Furthermore, medication rationing (skipping doses) was not considered in this analysis. However, results of a sensitivity analysis using a window of 90 days were highly similar to those of our primary analysis. Third, the proportion of patients taking tirzepatide was small given the timing of the study and its relatively more recent approval. Fourth, mild GI adverse events (eg, nausea and vomiting) were not included in the analysis, given an expectation of inconsistent capture in the EHR. Fifth, many patients who discontinued were excluded from reinitiation analyses because they did not have a weight measurement within 60 days of the discontinuation date. Patients without a follow-up visit and/or weight measurement around the time of discontinuation may be different from patients with weight measurements available.

## Conclusions

In this cohort study of adults with overweight or obesity who initiated GLP-1 RA therapy, most discontinued therapy within 1 year. Those without type 2 diabetes discontinued their GLP-1 RA at a significantly higher rate and reinitiated a GLP-1 RA at a significantly lower rate compared with those with type 2 diabetes. Greater weight loss and higher income (type 2 diabetes only) were associated with lower discontinuation, while weight regain since discontinuation was associated with higher reinitiation. Access to and insurance coverage of GLP-1 RAs for patients without type 2 diabetes may have been associated with these differences. Inequities in access and adherence to effective treatments have the potential to exacerbate disparities in obesity.

## Notes

Supplement 1.
eMethods.

eResults.
**eFigure 1.** Results of Alternative Models That Include Time-Varying Coefficients on Non-Proportional Hazards for Discontinuation **eFigure 2.** Results of Alternative Models That Include Time-Varying Coefficients on Non-Proportional Hazards for Reinitiation **eFigure 3.** Results of Discontinuation Analyses Using an Alternative Discontinuation Window of 90 Days **eFigure 4.** Results of Reinitiation Analyses Using an Alternative Discontinuation Window of 90 Days **eFigure 5.** Discontinuation Reasons Extracted From Clinical Notes, Among the Subpopulation With Both Dispense-Based and Notes-Based Discontinuation Within 365 Days

Supplement 2.
Data Sharing Statement
