# Supplementary material for: Discontinuation and Reinitiation of Dual-Labeled GLP-1 Receptor Agonists Among US Adults With Overweight or Obesity
Source: JAMA Netw Open. 2025 Jan 31;8(1):e2457349. doi: 10.1001/jamanetworkopen.2024.57349 (PMC11786232; doi:10.1001/jamanetworkopen.2024.57349)
Supplement: Supplement 1. — eMethods. eResults. eFigure 1. Results of Alternative Models That Include Time-Varying Coefficients on Non-Proportional Hazards for Discontinuation eFigure 2. Results of Alternative Models That Include Time-Varying Coefficients on Non-Proportional Hazards for Reinitiation eFigure 3. Results of Discontinuation Analyses Using an Alternative Discontinuation Window of 90 Days eFigure 4. Results of Reinitiation Analyses Using an Alternative Discontinuation Window of 90 Days eFigure 5. Discontinuation Reasons Extracted From Clinical Notes, Among the Subpopulation With Both Dispense-Based and Notes-Based Discontinuation Within 365 Days [file jamanetwopen-e2457349-s001.pdf]

Supplementary Online Content

Rodriguez PJ, Zhang V, Gratzl S, et al. Discontinuation and reinitiation of dual-labeled GLP-1 receptor agonists among US adults with overweight or obesity. *JAMA Netw Open.* 2025;8(1):e2457349. doi:10.1001/jamanetworkopen.2024.57349

eMethods.

eResults.

eFigure 1. Results of Alternative Models That Include Time-Varying Coefficients on Non-Proportional Hazards for Discontinuation

eFigure 2. Results of Alternative Models That Include Time-Varying Coefficients on Non-Proportional Hazards for Reinitiation

eFigure 3. Results of Discontinuation Analyses Using an Alternative Discontinuation Window of 90 Days

eFigure 4. Results of Reinitiation Analyses Using an Alternative Discontinuation Window of 90 Days

eFigure 5. Discontinuation Reasons Extracted From Clinical Notes, Among the Subpopulation with Both Dispense-Based and Notes-Based Discontinuation Within 365 Days

This supplementary material has been provided by the authors to give readers additional information about their work.

eMethods

Model-fitting procedures

Survival methods were used to accommodate real-world data subject to right censoring. This also allowed for the inclusion of tirzepatide, which was approved less than 2 years before the study end. In all analyses, patients were censored at the first of: 1) their last encounter, 2) 2 years from their index event, or 3) study end (November 5, 2024).

GLP-1 RA initiation (first medication dispense) served as the index event for discontinuation analyses. Discontinuation was defined as a 60-day gap in medication availability, accounting for excess supply from previous medication fills. GLP-1 RA discontinuation served as the index event for reinitiation analyses. Reinitiation was defined as the first GLP-1 RA fill after discontinuation.

Time-varying Cox proportional hazards models for discontinuation were fit using pre-specified variables of interest a priori. Demographic and social drivers of health (SDOH) variables were age, sex, race, and individual income. Age at initiation was modeled as both a continuous variable and a binary indicator of age greater than or equal to 65, a proxy for Medicare age eligibility, given Medicare's uniquely restrictive policies on coverage of anti-obesity medications (AOMs). Health factors included baseline body mass index (BMI), and the comorbidities of chronic kidney disease (CKD) and heart failure (HF). Incident moderate or severe GI adverse events (AEs) were modeled as time-varying covariates, which had a value of zero until the first date they appeared in the EHR. Weight loss on treatment was included as a time-varying covariate that was updated each time a new weight value was recorded and modeled as percentage change relative to baseline. If no weight measurements were taken following initiation, this value remained zero until censoring or discontinuation. This time-varying covariate assumes a stepwise weight change, where weight values do not change between visits. Finally, baseline hazards were stratified by index year, to account for changing patterns in use and to partially account for differences in availability of medications over time. Separate models were fit for patients with and without type 2 diabetes (T2D), given the expectation that several factors may differ between these groups, particularly given differences in insurance coverage of patients without T2D.

Time-varying Cox proportional hazards models for reinitiation were fit using a similar approach, with a few deviations. First, both presence of any moderate or severe GI AE during initial treatment and total weight loss on treatment were included as time invariant covariates in the reinitiation models. Total weight loss on treatment was the difference between baseline and the last value on treatment, modeled as a percentage change. Second, duration of initial treatment (in months) was included as a time invariant covariate. Finally, weight gain since discontinuation was included as a time-varying covariate. As described above, the covariate value was updated each time a new weight was recorded after discontinuation. For interpretability, this was modeled per 1% gain relative to the discontinuation weight.

© 2025 Rodriguez PJ et al. *JAMA Network Open.*

In all models, proportional hazards were tested using scaled Schoenfeld residuals over time. In both analyses, violations of proportional hazards occurred (age, weight loss, and baseline BMI), indicating the association between the predictor and outcome (either discontinuation or reinitiation) were not constant over time. To account for time-varying relationships between predictors and outcomes, secondary models included interactions with time strata, allowing for changes in the coefficient in each 6-month increment. Across all models, coefficient effect estimates were similar in models with and without time interactions (eFigures 1 and 2). Given similarity of estimates and the desire for interpretability, primary models presented in this paper are for time invariant coefficients.

Clinical Notes Analysis

Clinical notes data were available for a subset of patients in Truveta data. GLP-1 adherence status (whether the patient stopped or was still taking the medication) and reasons for discontinuation (why the patient stopped, if documented) were extracted from clinical notes by Truveta using natural language processing (NLP). In a validation sample of 350 GLP-1 discontinuation notes that were evaluated by clinical terminologists, NLP model precision was 88.1%, recall was 89.8%, and accuracy was 80.0%. Reasons for discontinuation extracted from clinical notes were characterized for those with a note within 365 days of the dispense-based discontinuation date.

© 2025 Rodriguez PJ et al. *JAMA Network Open.*

eResults
Time varying coefficients

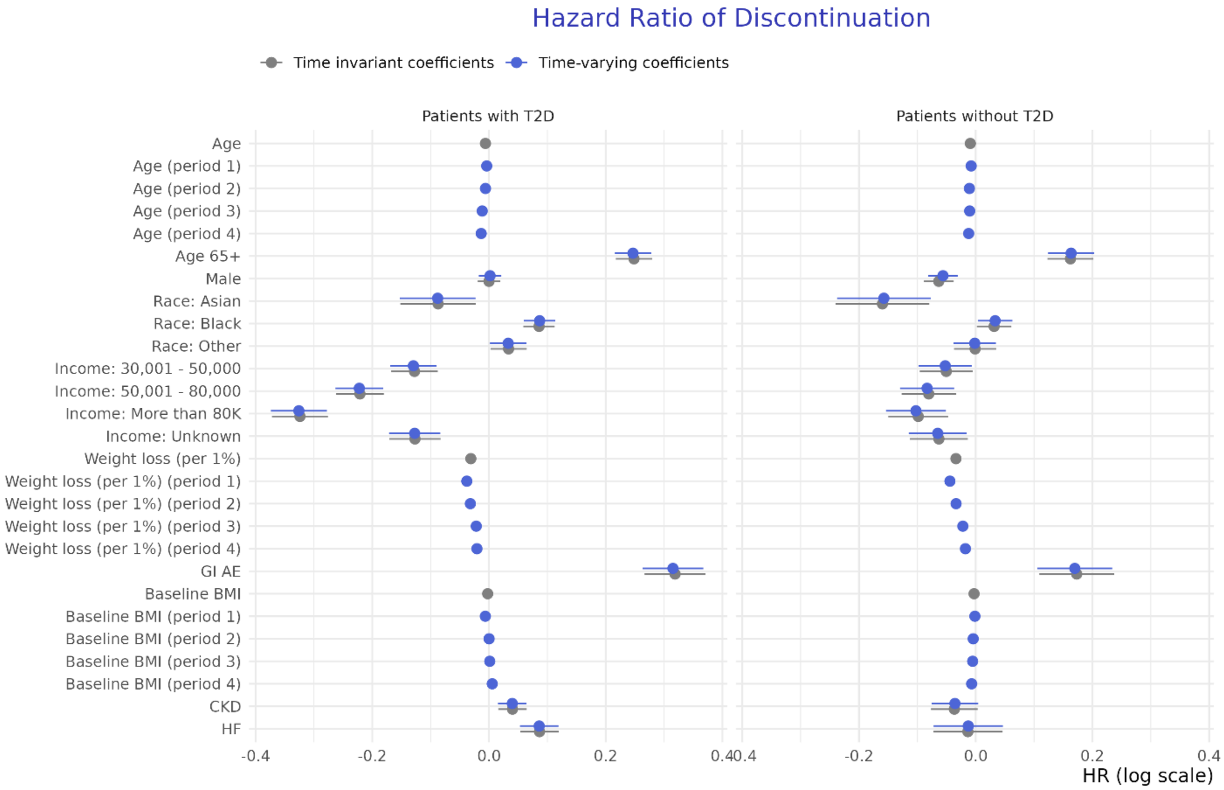

eFigure 1. *Results of Alternative Models That Include Time-Varying Coefficients on Non-Proportional Hazards for Discontinuation. Estimates are presented as hazard ratios on a log scale. Grey points represent the primary, time-invariant coefficients presented in the paper. Blue dots represent alternative, time-varying coefficients. The reference categories for sex, race, and income were female, white, and ≤$30,000, respectively. Periods correspond to 6-month periods from initiation (e.g., 0-6 months, 6-12 months, 12-18 months, 18-24 months). Baseline hazards were stratified by initiation year.*

© 2025 Rodriguez PJ et al. *JAMA Network Open.*

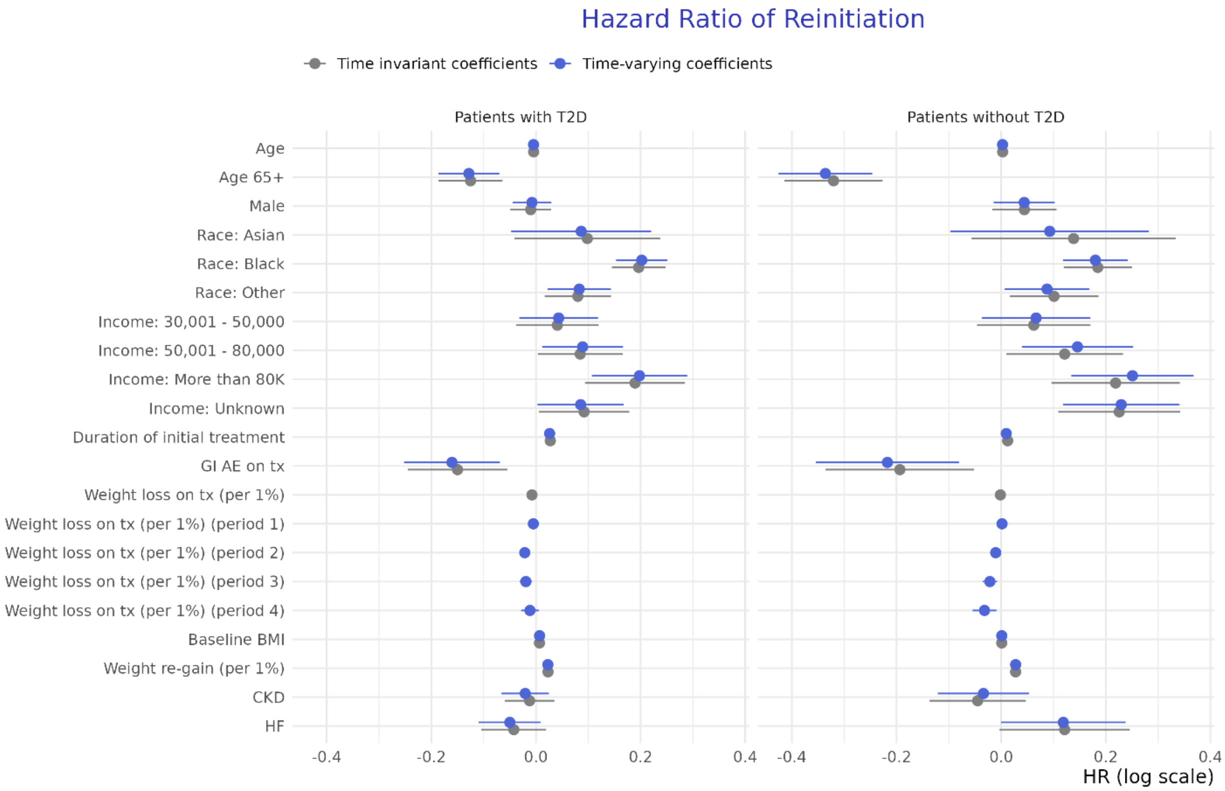

eFigure 2. *Results of Alternative Models That Include Time-Varying Coefficients on Non-Proportional Hazards for Reinitiation. Estimates are presented as hazard ratios on a log scale. Grey points represent the primary, time-invariant coefficients presented in the paper. Blue dots represent alternative, time-varying coefficients. The reference categories for sex, race, and income were female, white, and ≤$30,000, respectively. Duration is measured per 30 days of initial treatment. Periods correspond to 6-month periods from initiation (e.g., 0-6 months, 6-12 months, 12-18 months, 18-24 months). Baseline hazards were stratified by initiation year.*

90-day discontinuation window

The proportion of patients discontinuing GLP-1 RA was lower in sensitivity analyses using a 90-day window, rather than a 60-day window. For patients with T2D, 39.3% (95% confidence interval [CI], 38.9% - 39.6%) discontinued by 1 year and 57.6% (57.2% - 58.0%) discontinued by 2 years using a 90-day window, compared to 46.5% (46.2% - 46.9%) by 1 year and 64.1% (63.7% - 64.5%) by 2 years using a 60-day window. For patients without T2D, 57.8% (57.4% - 58.3%) discontinued by 1 year and 80.5% (80.1% - 81.0%) by 2 years using a 90-day window, compared to 64.8% (64.4% - 65.2%) by 1 year and 84.4% (84.0% - 84.8%) by 2 years using a 60-day window.

© 2025 Rodriguez PJ et al. *JAMA Network Open.*

Not surprisingly, reinitiation rates were lower when using a longer discontinuation window. For patients with T2D, 40.3% (39.6% - 41%) and 51.7% (50.8% - 52.6%) reinitiated by 1 and 2 years, respectively, using a 90-day window, compared to 47.3% (46.6% - 48.0%) and 57.3% (56.5% - 58.1%) using a 60-day window. For patients without T2D, 31.4% (30.7% - 32.2%) and 42.5% (41.4% - 43.6%) reinitiated by 1 and 2 years, respectively, using a 90-day window, compared to 36.3% (35.6% - 37%) and 46.4% (45.4% - 47.4%) using a 60-day window.

Although the proportions of discontinuation varied between the 60- and 90-day windows, hazard ratios for factors associated with discontinuation and reinitiation outcomes were consistent across both timeframes, with overlapping confidence intervals (eFigures 3 and 4).

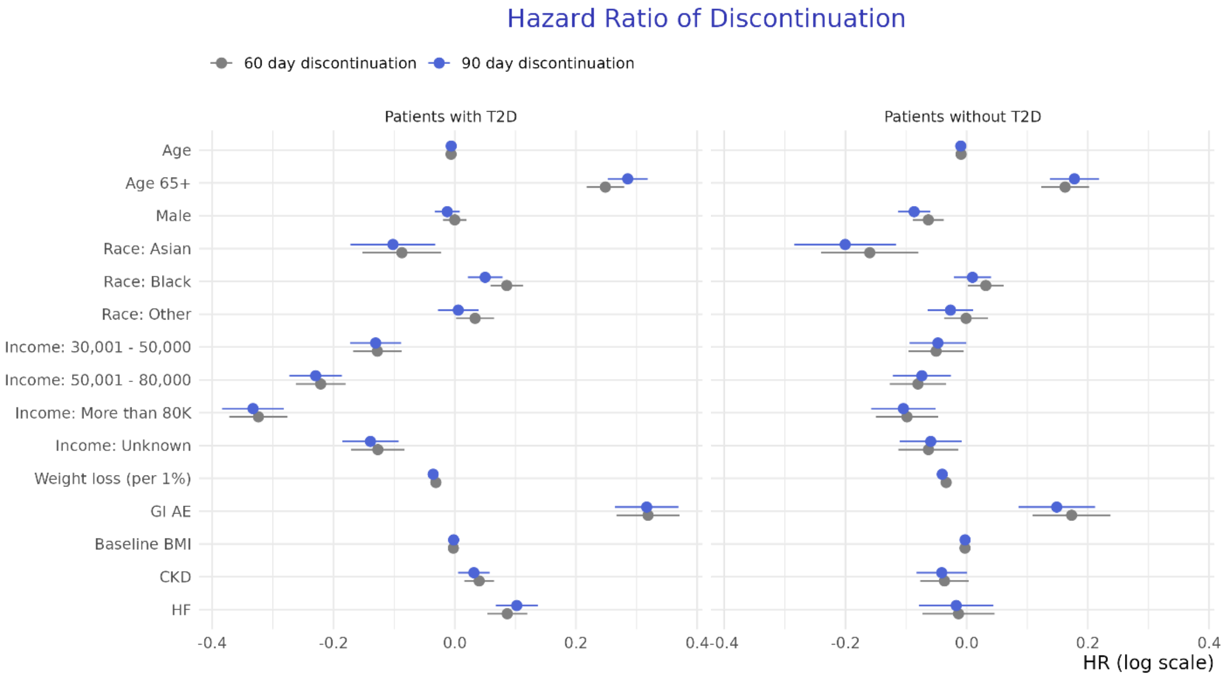

eFigure 3. *Results of Discontinuation Analyses Using an Alternative Discontinuation Window of 90 Days. Estimates are presented as hazard ratios on a log scale. Grey points represent the primary coefficients presented in the paper, using a 60-day window for discontinuation. Blue dots represent alternative estimates using a 90-day window for discontinuation. The reference categories for sex, race, and income were female, white, and ≤$30,000, respectively. The reference categories for sex, race, and income were female, white, and ≤$30,000, respectively. Baseline hazards were stratified by initiation year.*

© 2025 Rodriguez PJ et al. *JAMA Network Open.*

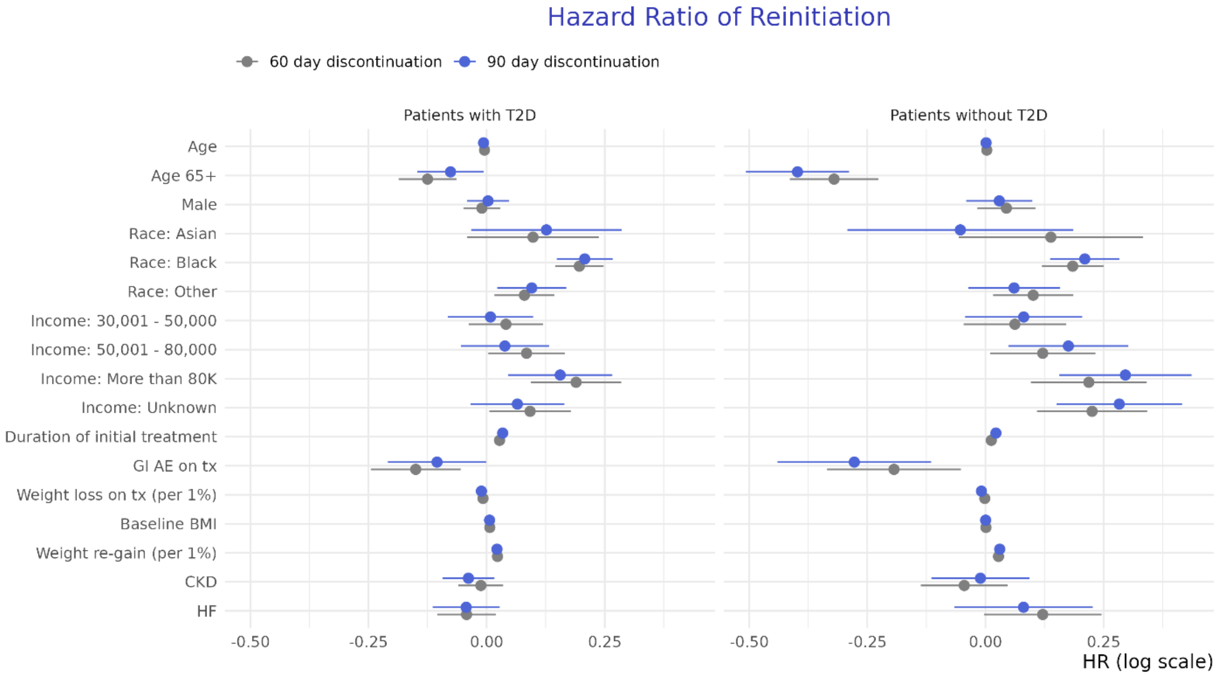

eFigure 4. *Results of Reinitiation Analyses Using an Alternative Discontinuation Window of 90 Days. Grey points represent the primary coefficients presented in the paper, using a 60-day window for discontinuation. Blue dots represent alternative estimates using a 90-day window for discontinuation. The reference categories for sex, race, and income were female, white, and ≤$30,000, respectively. Duration is measured per 30 days of initial treatment. The reference categories for sex, race, and income were female, white, and ≤$30,000, respectively. Baseline hazards were stratified by initiation year.*

Clinical notes analyses

For the study patients with a clinical note documenting an adherence status of ‘stopped’, ‘on hold’, or ‘not taking’ for their index GLP-1 RA, 77.3% were also identified as discontinued using dispense-based methods with a window of 60 days. This increased with a shorter window for dispense-based discontinuation (84.3% with a 30-day window) and decreased when using a longer window (72.1% with a 90-day window).

© 2025 Rodriguez PJ et al. *JAMA Network Open.*

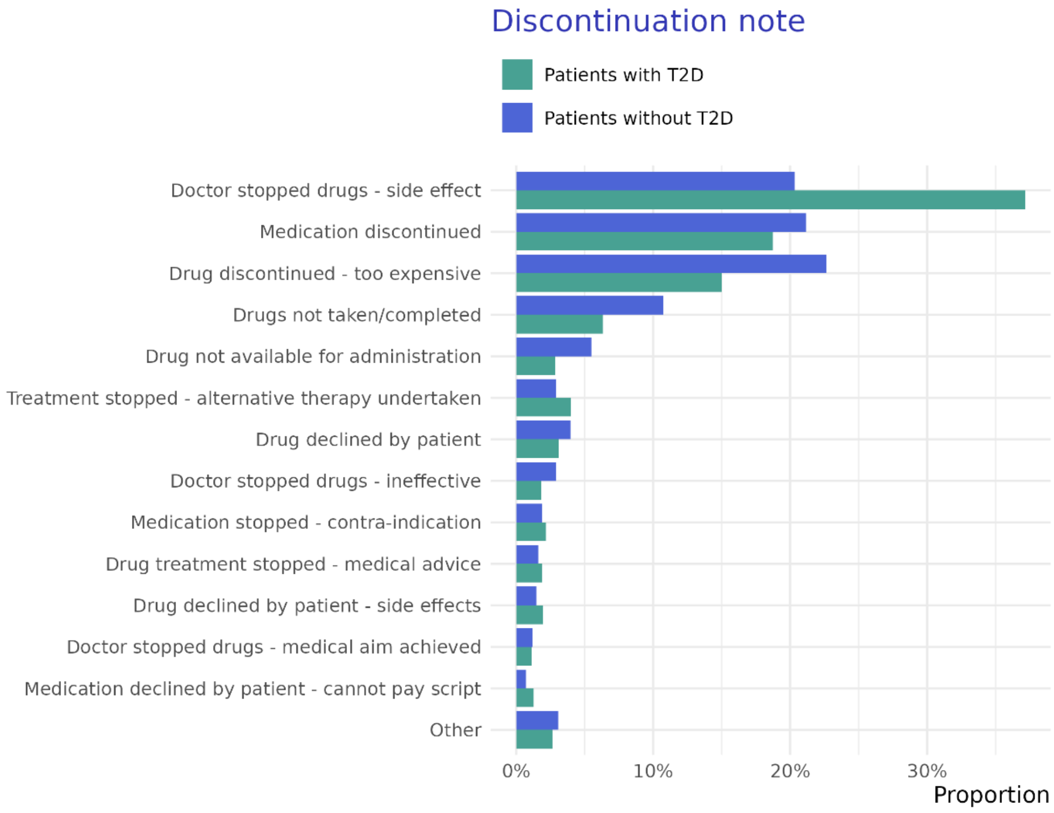

eFigure 5. *Discontinuation Reasons Extracted From Clinical Notes, Among the Subpopulation with Both Dispense-Based and Notes-Based Discontinuation Within 365 Days. Proportions represent the proportion of patients in each T2D strata with the discontinuation reason in extracted clinical notes. A single note was used per patient.*

© 2025 Rodriguez PJ et al. *JAMA Network Open.*
