# Supplementary material for: Discontinuation and Reinitiation of Dual-Labeled GLP-1 Receptor Agonists Among US Adults With Overweight or Obesity
Source: JAMA Netw Open. 2025 Jan 31;8(1):e2457349. doi: 10.1001/jamanetworkopen.2024.57349 (PMC11786232; doi:10.1001/jamanetworkopen.2024.57349)
Supplement: Supplement 2. — Data Sharing Statement [file jamanetwopen-e2457349-s002.pdf]

Data Sharing Statement

Rodriguez. Discontinuation and Reinitiation of Dual-Labeled GLP-1 Receptor Agonists Among US Adults With Overweight or Obesity. *JAMA Netw Open*. Published January 31, 2025. doi:10.1001/jamanetworkopen.2024.57349

Data
Data available: No
Explanation for why data are not available: Data are not publicly available. Truveta data are available to Truveta subscribers.
